# Impact of *STAT6* Variants on the Response to Proton Pump Inhibitors and Comorbidities in Patients with Eosinophilic Esophagitis

**DOI:** 10.3390/ijms25073685

**Published:** 2024-03-26

**Authors:** Paula Soria-Chacartegui, Marcos Navares-Gómez, Francisca Molina-Jiménez, Emilio J. Laserna-Mendieta, Laura Arias-González, Pedro Majano, Sergio Casabona, Alfredo J. Lucendo, Francisco Abad-Santos, Cecilio Santander, Pablo Zubiaur

**Affiliations:** 1 Clinical Pharmacology Department, Hospital Universitario de La Princesa, Faculty of Medicine, Universidad Autónoma de Madrid (UAM), 28006 Madrid, Spain; 2 Instituto de Investigación Sanitaria La Princesa (IIS-IP), 28006 Madrid, Spainajlucendo@hotmail.com (A.J.L.); 3 Gastroenterology Department, Hospital Universitario de La Princesa, Faculty of Medicine, Universidad Autónoma de Madrid (UAM), 28006 Madrid, Spain; 4 Centro de Investigación Biomédica en Red de Enfermedades Hepáticas y Digestivas (CIBERehd), Instituto de Salud Carlos III, 28029 Madrid, Spain; 5 Gastroenterology Department, Hospital General de Tomelloso, 13700 Ciudad Real, Spain; 6 Instituto de Investigación Sanitaria de Castilla-La Mancha (IDISCAM), 45071 Toledo, Spain; 7 Cellular Biology Department, Faculty of Biology, Universidad Complutense de Madrid, 28040 Madrid, Spain

**Keywords:** *STAT6*, pharmacogenetics, eosinophilic esophagitis, *CYP2C19*

## Abstract

Proton pump inhibitors (PPIs) are the first-line drug for eosinophilic esophagitis (EoE), although it is estimated that there is a lack of histological remission in 50% of patients. This research aimed to identify pharmacogenetic biomarkers predictive of PPI effectiveness and to study their association with disease features. Peak eosinophil count (PEC) and the endoscopic reference score (EREFS) were determined before and after an eight-week PPI course in 28 EoE patients. The impact of the signal transducer and activator of transcription 6 (*STAT6*), *CYP2C19*, *CYP3A4*, *CYP3A5,* and *ABCB1* genetic variations on baseline PEC and EREFS, their reduction and histological response, and on EoE symptoms and comorbidities was analyzed. PEC reduction was higher in omeprazole-treated patients (92.5%) compared to other PPIs (57.9%, *p* = 0.003). *STAT6* rs12368672 (g.18453G>C) G/G genotype showed higher baseline PEC values compared to G/C and C/C genotypes (83.2 vs. 52.9, *p* = 0.027). EREFS reduction in *STAT6* rs12368672 G/G and G/C genotypes was higher than in the C/C genotype (36.7% vs. −75.0% *p* = 0.011). However, significance was lost after Bonferroni correction. Heartburn incidence was higher in *STAT6* rs167769 (g.27148G>A) G/G patients compared to G/A (54.55% vs. 11.77%, *p* = 0.030). *STAT6* rs12368672G>C and rs167769G>A variants might have a relevant impact on EoE status and PPI response. Further research is warranted to clarify the clinical relevance of these variants.

## 1. Introduction

Eosinophilic esophagitis (EoE) is an immune-mediated disease characterized by an eosinophil-predominant inflammation restricted to the esophagus and by the appearance of symptoms of esophageal dysfunction [1]. It is considered a type 2 inflammatory response mainly triggered by food antigens [1,2]. In EoE, dietary allergens activate the esophageal epithelium and trigger the production of T-helper (Th)-2 cytokines that stimulate different immune cells. Interleukin (IL)-4 and IL-13 locally produced activate the signal transducer and activator of transcription 6 (STAT6), which induces eotaxin-3 (also called *CCL26*) expression. Eotaxin-3 promotes the recruitment of eosinophils from blood to esophageal tissues, leading to eosinophilic inflammation [3]. Additionally, lymphocytes in the esophagus produce other Th-2 cytokines, such as IL-5 and IL-15, that contribute to the perpetuation of inflammation, altering the barrier function, increasing esophageal permeability to dietary antigens, and promoting tissue remodeling [2], which in the long term results in fibrosis that is manifested as rings and strictures in endoscopy. These alterations determine a variety of symptoms that include dysphagia, food impaction, heartburn, regurgitation, vomiting, nausea, and abdominal pain [1,4].

Fist-line treatment options for EoE include dietary modifications, proton pump inhibitors (PPIs), and swallowed topical corticosteroids [4]. PPIs are widely accessible and convenient drugs, therefore representing the most commonly used therapy at all ages and in most settings [5,6,7]. The primary mechanism of action of PPIs is the blockage of the gastric H, K-ATPase, thereby inhibiting gastric acid secretion [8]. The CYP2C19 genotype-informed metabolic phenotype was shown to impact PPI pharmacokinetics, safety, and efficacy in several diseases, including peptic ulcer, gastroesophageal reflux disease, and *Helicobacter pylori* eradication. For this reason, the Clinical Pharmacogenetics Implementation Consortium (CPIC) has published a pharmacogenetic guideline recommending omeprazole, pantoprazole, and lansoprazole dose adjustments based on the CYP2C19 phenotype [9]. To further optimize and personalize EoE treatment with PPIs, other biomarkers have been proposed [10,11], albeit their clinical relevance has not yet been proven. One of these biomarkers is *STAT6*, which has been identified as a PPI target in EoE patients, as it is involved in drug pharmacodynamics since PPIs block STAT6 binding to the *CCL26* promoter [10]. Therefore, the aim of this research was to analyze the impact of *CYP2C19* and *STAT6* genetic variation (and variation in other relevant pharmacogenes such as *CYP3A4*, *CYP3A5,* or *ABCB1*) on PPI response, and of *STAT6* variants on EoE baseline status (i.e., peak eosinophils count, endoscopic phenotype, and symptoms) and comorbidities. It should also be noted that with *STAT6*, as with other pharmacogenes, there is a fine line between human genetics and pharmacogenetics; some biomarkers can be both diagnostic and pharmacogenetic. Thus, this research also aimed to understand the role of *STAT6* genetic variants in the development and progression of EoE. This research is part of the La Princesa Multidisciplinary Initiative for the Implementation of Pharmacogenetics (PriME-PGx) [12].

## 2. Results

### 2.1. Baseline Characteristics

Overall, 28 patients were included in this research. Women showed lower weight, height, and body mass index (BMI) compared to men (*p* = 0.007, *p* = 0.020 and *p* = 0.042, respectively), and similar age (*p* = 0.193) (Table 1). At least one symptom was present in every patient, with the majority having two symptoms (53.6%), followed by three (21.4%), five (10.7%), and four symptoms (7.1%). Only one patient had one symptom (3.6%), and one patient had six symptoms (3.6%). The majority of patients included in this research suffered atopic diseases (25 out of 28, 89.3%), with two patients showing three different clinical manifestations (7.1%), fourteen patients showing two (50%), and nine presenting one (32.1%), whereas three patients did not suffer atopic diseases (10.7%).

Omeprazole was the PPI most frequently administered (53.57%), followed by pantoprazole (16.86%), lansoprazole, and esomeprazole (14.29% each). Baseline scores and disease duration were unrelated to sex or treatment (Table 2). However, a mean difference of 8.5 years of disease duration was observed between patients treated with omeprazole vs. esomeprazole (*p* = 0.080) (Table 2).

### 2.2. Impact of STAT6 Genetic Variation in Baseline Scores

A higher baseline peak eosinophil count (PEC) was observed in patients with the *STAT6* rs12368672 G/G genotype compared to those with the G/C + C/C genotypes (*p* = 0.027); likewise, a trend towards higher baseline exudates, rings, edema, furrows, and stricture (EREFS) scale (or endoscopic reference scale) was observed in patients with the *STAT6* rs12368672 G/G + G/C genotypes compared to those with the C/C genotype (*p* = 0.086) (Table 2). However, significance was not reached after Bonferroni correction for multiple comparisons (*p*-value established at *p* < 0.004). No further differences in baseline scores and disease duration according to *STAT6* genotypes were observed (Table 2).

### 2.3. Impact of STAT6 Genetic Variation on Symptom Onset and Comorbidity

Heartburn incidence was higher in patients with the *STAT6* rs167769 G/G genotype compared to G/A (54.55% vs. 11.77%, *p* = 0.030) (Table 3). Also, a tendency towards higher heartburn incidence in the *STAT6* rs324011 G/G genotype compared to G/A + A/A genotypes (50.00% vs. 16.67%, *p* = 0.091), and in the *STAT6* rs12368672 G/G genotype compared to the G/C + C/C genotypes (46.15% and 13.33%, *p* = 0.096), was observed (Table 3).

A tendency towards lower food allergy incidence in patients with the G/G + G/C genotypes for *STAT6* rs12368672 compared to those with the C/C genotype (*p* = 0.074), and a tendency towards higher asthma incidence in carriers of the *STAT6* rs324015 A/G genotype compared to those with the G/G genotype (*p* = 0.097), were observed (Table 3).

### 2.4. Treatment Effectiveness

Fifteen out of the 28 patients (53.6%) were classified as responders; compared to non-responders, they showed lower mean ± standard deviation baseline PEC (53.67 ± 30.42 versus 82.62 ± 52.44, *p* = 0.038), EREFS (3.67 ± 1.95 versus 4.85 ± 1.68, *p* = 0.101), and disease duration (2.00 ± 10.00 versus 6.00 ± 22.50 years, *p* = 0.142). Overall, PEC decreased in 100% of responders and in 53.8% of non-responders, it remained unchanged in 15.4% of non-responders, and it increased in the remaining 30.8% (*p* = 0.005). A decrease in EREFS score was observed in 80.0% of responders and 46.1% of non-responders; it increased in 6.7% and 23.1%, and did not change in 13.3% and 30.8%, respectively (*p =* 0.211). PEC and EREFS score reductions were also significantly higher in responders compared to non-responders (*p* < 0.001 and *p* = 0.005, respectively) (Table 4). The three women who participated in the study were classified as responders, compared to 48% of men (n = 12 out of 25) (*p* = 0.226). 

PEC reduction was higher among patients treated with omeprazole compared to the remaining ones (i.e., esomeprazole, pantoprazole, and lansoprazole, *p* = 0.003), and was also related to a higher response rate (74%), followed by lansoprazole (50%), esomeprazole (25%), and pantoprazole (20%) (*p* = 0.125) (Table 4). However, when including disease duration, differences in treatment effect on PEC reduction disappeared. No differences were observed in EREFS reduction according to treatment, nor in EREFS and PEC reduction according to sex. 

### 2.5. Correlation between Severity Scores

Positive correlations between baseline PEC and EREFS score (*p* < 0.001, r = 0.640), between baseline EREFS and EREFS reduction (*p* = 0.042, r = 0.388), and between PEC reduction and EREFS reduction (*p* = 0.034, r = 0.403) was observed. In contrast, no correlation between baseline PEC and PEC reduction was observed (*p* = 0.994). A higher EREFS score reduction was associated with lower disease duration (*p* = 0.006, r = −0.507). A negative trend between PEC reduction and disease duration was observed (*p* = 0.062, r = −0.357), with no correlation between disease duration and baseline PEC or baseline EREFS (*p* = 0.771 and *p* = 0.398, respectively).

### 2.6. Impact of Genetic Variation on Effectiveness Variables and Histological Response

Patients with the *STAT6* rs12368672 C/C genotype showed a lower reduction in EREFS score compared to patients with G/C + G/G genotypes (*p* = 0.011) (Table 5). Furthermore, a higher EREFS score reduction was observed in individuals with *ABCB1* rs2032582 T/T+T/G genotypes compared to those with A/A+G/A+G/G genotypes (*p* = 0.045) (Table 5); none of these differences reached the threshold for statistical significance after the Bonferroni correction for multiple comparisons (*p*-value established at *p* < 0.004). Regarding CYP2C19, no significant differences in PEC or EREFS score reduction were observed, although an approximately 35% lower PEC reduction was observed in rapid metabolizers (RM) compared to normal, intermediate, and poor metabolizers (NM, IM and PM, respectively) (*p =* 0.359). No significant differences were observed for PEC or EREFS score reduction according to the remaining genotypes (Table 5). No associations between genotypes or phenotypes and histological response were found (Table S1).

### 2.7. Impact of Genetic Variation on Symptom Variations

All patients with the *CYP3A4* *1/*1 genotype suffered dysphagia (n = 26), which disappeared in seven of them after treatment (26.92%), compared to one out of the two patients with the *CYP3A4* *1/*22 genotype, in whom it also disappeared (*p* = 0.021). Fifteen patients with the *STAT6* rs1059513 A/A genotype (71.42%) suffered from food impaction; in fourteen of them it improved after treatment (93.33%) compared to the seven patients with the *STAT6* rs1059513 A/G genotype who suffered food impaction (100%); in three of them (42.85%), the symptom was relieved (*p* = 0.011). No further association in symptom changes and genotypes or phenotypes were found.

## 3. Discussion

PPIs are the most widely used first-line treatment for EoE due to their safety profile, easy administration, and low cost [5,6]. However, it is estimated that only 50% of patients under PPI treatment reach histological remission [13], and personalizing pharmacological therapy might be a key tool to increase this rate of response. Thus, the objective of this research was to find useful predictors of PPI response in patients with EoE, which might be useful not only to guide drug selection but also dose optimization, which is common and highly relevant in the treatment of EoE with PPIs.

Eighty-nine percent of patients included in this research were men (8:1 ratio), which is concordant with the well-defined higher incidence of EoE among males [14,15,16]. As in other series [17], omeprazole was the most commonly used PPI (53.6%), and it led to higher PEC reduction compared to other active ingredients. These results are partially consistent with previous research, in which a trend towards a higher omeprazole and esomeprazole effectiveness was observed [17]. In Spain, the cost for an omeprazole 20 mg capsule is EUR 0.09, compared to EUR 0.45, 0.57, and 0.62 for esomeprazole 20 mg, lansoprazole 30 mg, and pantoprazole 40 mg, respectively [18]. This five- to seven-fold price difference might justify omeprazole predominance. The better response observed in patients treated with omeprazole may be explained by their lower disease duration, as longer disease duration is associated with a transition to a fibrotic state, resulting in a lower response to pharmacological therapy [19].

Several studies have shown the role of STAT6 in the eosinophilic inflammation of EoE by inducing *CCL26* expression [3]. However, its role as a pharmacogenetic biomarker in this disease and the association with its symptoms and comorbidities have not been widely studied. In this work, the g.18453G>C far upstream variant in *STAT6* (rs12368672) was related to lower baseline PEC and to lower EREFS score reduction. In a previous article, an association between this variant and higher eosinophil/hpf levels previous to PPI treatment was found [11]. Although these results appear to be contradictory, it should be taken into account that *STAT6* is located in the reverse strand of DNA, the probe used in both studies (C_31186828_10, Applied Biosystems, Thermofisher, Waltham, MA, USA) provides the genotype in the forward strand, and this variant entails a change between complementary nucleotides, which might act as a cofounding factor [20]. Thus, a clear description of the nomenclature and reference sequence is needed to enable comparison and conclusion drawing. Additionally, differences in the study populations (adult versus pediatric) should also be considered. In addition, three articles have supported the relevance of this variant in food allergy, which is consistent with the trend observed in our work towards a higher prevalence of food allergy in patients with the *STAT6* g.18453G>C (rs12368672) C/C genotype compared to the G/G and G/C genotypes [21,22,23]. The lack of a clear description of the reference sequence used, as mentioned above, prevents us from jointly weighing the direction of the association. Nevertheless, the fact that different and independent articles have reported an effect of the same *STAT6* variant suggests its potential relevance, not only in EoE but in its comorbidities and other allergic or atopic diseases. Additionally, patients with the *STAT6* g.27148G>A (rs167769) G/G genotype suffered from heartburn with higher frequency than patients with the G/A genotype, and a similar trend was observed for the g.28741G>A (rs324011) and g.18453G>C (rs12368672) G/G genotypes, probably due to its high, although not complete, linkage disequilibrium with rs167769 [11]. Lastly, patients with the *STAT6* g.41214A>G (rs1059513) A/A genotype had a higher frequency of food impaction improvement after treatment compared to those with the A/G genotype. To our knowledge, this is the first work to find such associations. Although these results should be considered cautiously, they suggest the relevance of *STAT6* genetic variation in EoE baseline status, symptoms, and comorbidities, shedding some light onto its impact on EoE mechanism of action. Due to the fine line between human genetics and pharmacogenetics, these results may open a way to predicting the risk of EoE development, thus facilitating early diagnosis, which would possibly lower the progression to a fibrotic phenotype, and the discovery of new targets for the treatment of this illness. 

In our research, PEC reduction was approximately 35% lower in CYP2C19 RMs compared NMs, IMs, and PMs, although this difference was not statistically significant, likely due to the reduced sample size. However, CPIC only considers dose adjustments for ultrarapid metabolizers (UMs) [9], a phenotype absent in our study and which shows even higher enzymatic activity than that of RMs; therefore, greater differences with respect to NMs, IMs, and PMs could be expected. Further research with increased sample sizes and including UMs is needed to assess whether CYP2C19 RMs may also benefit from a PPI dose increase in EoE treatment, as shown in a different study [24].

Lastly, PPIs are proposed to be substrates and inhibitors of the *ABCB1*-coded transporter, P-glycoprotein (P-gp) [25]. In this research, a nominal association between the *ABCB1* g.186947T>G/A (rs2032582) G/G, G/A, and A/A genotypes and lower EREFS reduction was observed compared to the T/T and T/G genotypes. Concordantly, these *ABCB1* genetic variants were found to alter PPI pharmacokinetics or pharmacodynamics in previous studies [26,27]. However, *ABCB1* structural and functional characterization is required prior to concluding the clinical relevance of variant–phenotype associations.

This study is intended as an exploratory and descriptive study, where statistical significance does not imply clinical relevance, especially in light of the limited sample size. Therefore, the main limitation of this study was the small sample size available, especially for the variants with a low prevalence within the population, which reduces the statistical power. This, along with the low incidence of ADRs associated with PPI treatment at standard doses [28,29], led to the lack of meaningful conclusions in the analysis of drug tolerability. Nevertheless, further research is needed on the safety of long-term treatment with high-dose PPIs [30,31] and on the ability of *STAT6* genetic variants to predict this response. In addition, a better analysis of the linkage disequilibrium between *STAT6* variants should be performed, which might lead to allele and posterior phenotype definition, and a clear description of the reference sequence used is also needed to allow for comparison between results [32,33]. Furthermore, a functional characterization of *STAT6* variants (i.e., their impact on STAT6 expression and/or function) would also be of interest to better predict their clinical consequences. Thus, further research is warranted to clarify the impact and clinical relevance of these associations, not only in adult patients but also in pediatric EoE patients. However, this study also has some strengths. It is a prospective study analyzing the main candidate genes for PPI response and EoE development in a population representative of the real EoE population, and in which variability was reduced by standardizing PPI treatment, duration, and dose. Additionally, this research also sheds some light on the impact of *STAT6* genetic variation on the EoE mechanism of action, which might open an avenue to its study as a diagnostic and pharmacogenetic biomarker.

## 4. Materials and Methods

### 4.1. Study Population and Procedures

This was an observational prospective study on 28 patients with newly diagnosed EoE according to current criteria [14] at two Spanish hospitals: Hospital Universitario de La Princesa (Madrid, Spain) and Hospital General de Tomelloso (Ciudad Real, Spain). They routinely attended the gastroenterology departments of either hospital as part of routine clinical practice between February 2018 and November 2020. They all gave informed consent to participate in the present study. The inclusion criteria were as follows: to be an adult patient newly diagnosed with EoE, and therefore naïve to EoE treatment, and to have been prescribed but not yet have started omeprazole, esomeprazole, lansoprazole, or pantoprazole treatment at least at double dose for eight weeks. The only exclusion criteria were pregnancy or lactation. Institutional review boards at both sites approved the study protocol.

At the baseline endoscopy, three esophageal biopsies were obtained from each proximal and distal esophagus for histopathological evaluation after hematoxylin and eosin staining. Esophageal eosinophilia was defined as an eosinophil count of ≥15 cells per high-power field (hpf) (corresponding to an area of 0.24 mm^2^) in one or more biopsy specimens at any esophageal level. The exclusion of other potential causes of the esophageal eosinophilia and the absence of eosinophilic infiltration in gastric and duodenal mucosa biopsies led to EoE diagnosis when symptoms of esophageal dysfunction were present [14,34]. In addition, three additional biopsies were collected at the mid esophageal third for investigational purposes. Patients underwent an eight-week period of PPI therapy with omeprazole, esomeprazole, lansoprazole, or pantoprazole. Drug selection was performed based on a physician–patient decision, and the dose administered was at a least double dose in every patient: omeprazole 40 or 80 mg daily, esomeprazole 40 or 80 mg daily, lansoprazole 60 mg daily, or pantoprazole 80 mg daily (Table S2). Endoscopy was repeated after an eight-week treatment. Endoscopic features were assessed by the EREFSscale [35], and PEC was counted in all esophageal biopsies. Percentage reductions from baseline PEC and EREFS to after an eighth-week PPI course were analyzed as effectiveness variables. Those patients that achieved histological response (i.e., less than 15 eosinophils per hpf in the biopsy) after PPI treatment were classified as responders, and those who did not (i.e., 15 or more eosinophils per hpf) were considered non-responders. Additionally, clinical data including demographics, symptoms, disease duration (defined from symptoms onset to baseline endoscopy), and atopic background were collected from all patients’ clinical records [36]. 

This study was approved by the Research Ethics Committee of the Hospital Universitario de La Princesa (PI17/0008, registration number 3107, 8 June 2017) and all subjects gave informed consent to participate. During the research, the Declaration of Helsinki, the Good Clinical Practice guidelines, and the Patient Autonomy Law (41/2002) were followed [37,38]. 

### 4.2. Esophageal Biopsies Processing, Genotyping, and Phenotyping

The three endoscopic biopsies obtained for research were collected and frozen under liquid N_2_ conditions and then disrupted with a mortar and pestle, grinding them to a fine powder used for DNA extraction with a NZYtech tissue genomic DNA isolation kit (BM13502) (NZYtech, Lisbon, Portugal).

Genes and variants related to PPI bioavailability (i.e., *CYP2C19*, *CYP3A4*, *CYP3A5, ABCB1*) or response (i.e., *STAT6)* and to the development of the disease (i.e., *STAT6*) were selected from the literature (Table 6). A QuantStudio 12K Flex instrument was used for genotyping. Eight *STAT6* variants were genotyped with TaqMan^®^ probes in a 96-Fast thermal block; twenty-one additional variants in four genes were genotyped with a custom OpenArray thermal block (Applied Biosystems, Thermofisher, Waltham, MA, USA) (Table 6). Star alleles were defined according to the PharmVar nomenclature website [32]. Genotype information was translated into phenotype in accordance with the CPIC guidelines for *CYP2C19* genotyping, PPI prescription [9], *CYP3A5*-tacrolimus [39], the PharmGKB/CPIC/PharmVar PGx Gene-specific information tables [40], and the Dutch Pharmacogenetic Working Group (DPWG) guideline for *CYP3A4* [41].

### 4.3. Statistical Analysis

Statistical analysis was performed with the SPSS software (version 23, SPSS Inc., Chicago, IL, USA). Outlier data were identified with Grubb’s test and excluded from the analysis (final sample size n = 28). Baseline status and effectiveness variable distributions were checked for normality with the Shapiro–Wilk test, and they were analyzed according to sex, treatment, histological response, and genotypes (for baseline status, only *STAT6* genotypes were considered). For normally distributed variables, a t-test or an ANOVA test followed by a Bonferroni post hoc test were used, depending on whether there were two, three, or more categories, respectively. For two-category variables that were not normally distributed, a Mann–Whitney U test was used, whereas a Kruskal–Wallis test was performed for not normally distributed variables with three or more categories. A Bonferroni correction for multiple comparisons was performed to control for type I error. The correlation between effectiveness variables, baseline scores, and disease duration was calculated with Pearson’s correlation coefficient. The Pearson coefficient (r) is shown for significant associations (*p* < 0.05). Additionally, a Chi^2^ or Fisher’s exact test were performed to search for associations between *STAT6* genetic variation and histological response (responders vs. non-responders) and symptoms and atopic disease incidence.

## 5. Conclusions

*STAT6* g.27148G>A (rs167769), g.18453G>C (rs12368672), and g.41214A>G (rs1059513) may have potential relevance as biomarkers that are predictive of EoE development and PPI response. However, their exact role on the disease and how it can be used to guide treatment require further investigation. 

## Figures and Tables

**Table 1:** Demographic characteristics and symptoms and atopic disease incidence.

Demographic Characteristics										
Variable		n	Age (Years)		Height (m)		Weight (kg)		BMI (kg/m^2^)	
			Mean	SD	Mean	SD	Mean	SD	Mean	SD
Sex	Men	25	42.24	13.33	1.79	0.09	81.94	13.00	25.62	3.15
	Women	3	31.67	31.67	1.66 *	0.03	59.33 *	9.29	21.55 *	2.56
Total		28	41.11	13.13	1.77	0.09	79.52	14.40	25.18	3.31
**Clinical characteristics**										
Symptom incidence			Dysphagia		27 out of 28 (96.43%)		Regurgitation		6 out of 28 (21.43%)	
			Food impaction		22 out of 28 (78.57%)		Chest pain		5 out of 28 (17.86%)	
			Heartburn		8 out of 28 (28.57%)		Abdominal pain		2 out of 28 (7.14%)	
			Vomiting		6 out of 28 (21.43%)		Nausea		2 out of 28 (7.14%)	
Atopic disease incidence			Allergic rhinoconjunctivitis		23 out of 28 (82.1%)		Food allergy		8 out of 28 (28.57%)	
			Asthma		9 out of 28 (32.14%)		Eczema		3 out of 28 (10.7%)	

Data shown as mean and standard deviation (SD). BMI: body mass index. * *p* < 0.05 vs. men.

**Table 2:** Baseline scores and disease duration according to sex, treatment, and *STAT6* variants.

Variable		N	Baseline PEC (Cells/Hpf)		Baseline EREFS (Score)		Disease Duration (Years)	
			Mean	SD	Mean	SD	Median	IQR
Sex	Men	25	68.56	45.39	4.32	1.95	3.00	0.00–10.50
	Women	3	55.00	30.41	3.33	1.15	1.00	-
Treatment	Omeprazole	15	59.67	51.74	3.73	2.09	1.00	0.00–10.00
	Esomeprazole	4	72.50	17.08	4.75	1.71	9.50	6.75–32.50
	Pantoprazole	5	62.00	26.83	4.00	1.41	3.00	1.50–17.50
	Lansoprazole	4	96.00	45.28	5.75	1.26	3.00	0.00–21.00
*STAT6* g.18453G>C (rs12368672)	G/G	13	83.23 *	50.86	4.62	1.50	3.00	0.00–18.00
	G/C	13	55.15	34.22	4.15	2.19	2.00	0.50–9.50
	C/C	2	40.00	0.00	2.00	0.00	10.50	-
*STAT6* g.27148G>A (rs167769)	G/G	11	66.46	28.20	4.18	1.25	2.00	0.00–10.00
	G/A	17	67.53	52.34	4.24	2.25	3.00	0.00–10.50
*STAT6* g.28741G>A (rs324011)	G/G	10	69.80	27.33	4.10	1.29	2.00	0.00–14.75
	G/A + A/A	18	65.61	51.43	4.28	2.19	4.50	0.50–10.50
*STAT6* g.37927C>T (rs841718)	C/C	3	62.67	21.94	3.33	0.58	2.00	-
	C/T	17	59.24	33.40	4.18	1.78	2.00	0.00–9.00
	T/T	8	85.50	64.55	4.63	2.45	6.00	0.50–13.75
*STAT6* g.38178C>T (rs3024974)	C/C	19	68.53	49.54	4.11	2.03	6.00	1.00–10.00
	C/T	9	64.11	30.60	4.44	1.67	1.00	0.00–20.00
*STAT6* g.40823A>G (rs324015)	A/G	9	61.67	35.00	3.78	1.20	6.00	2.00–15.00
	G/G	19	69.68	48.08	4.42	2.14	1.00	0.00–10.00
*STAT6* g.41214A>G (rs1059513)	A/A	21	63.38	47.22	3.95	1.96	2.00	0.00–10.00
	A/G	7	78.29	31.74	5.00	1.53	9.00	0.00–15.00
TOTAL		28	67.11	43.80	4.21	1.89	2.50	0.00–10.00

Data shown as mean and standard deviation (SD) for baseline MEC and baseline EREFS and as median and interquartile range (IQR) for disease duration. PEC: peak eosinophils count; EREFS: endoscopic reference score. Hpf: high-power field. * *p* < 0.05 vs. *STAT6* rs12368672 G/C + C/C (i.e., nominally significant); no association reached the significance threshold after Bonferroni correction for multiple comparisons. All patients showed the *STAT6* rs2598483 G/G genotype. Variants were mapped using the *STAT6* NG_021272.2 RefSeqGene (LRG_1369) reference sequence.

**Table 3:** Differences in symptom onset and comorbidity incidence according to *STAT6* variants.

*STAT6* Variant	Genotype	Symptom/Comorbidity	Patients Affected	Significance
g.27148G>A (rs167769)	G/G	Heartburn	6 of 11 (54.55%)	*p* = 0.030
	G/A		2 of 17 (11.77%)	
g.18453G>C (rs12368672)	G/G		6 of 13 (46.15%)	*p* = 0.096
	G/C		1 of 13 (7.69%)	
	C/C		1 of 2 (50%)	
g.28741G>A (rs324011)	G/G		5 of 10 (50%)	*p* = 0.091
	G/A + A/A		3 of 18 (16.67%)	
g.18453G>C (rs12368672)	G/G	Food allergy	4 of 13 (30.8%)	*p* = 0.065
	G/C		2 of 13 (15.4%)	
	C/C		2 of 2 (100%)	
g.40823A>G (rs324015)	A/G	Asthma	5 of 9 (55.6%)	*p* = 0.097
	G/G		4 of 19 (21.1%)	

**Table 4:** Percentage of PEC reduction and EREFS score reduction from baseline to after eight-week treatment according to histological response, sex, and treatment.

Variable		N	PEC Reduction %		EREFS Score Reduction %	
			Median	IQR	Median	IQR
Histological response	Responders	15	99.23	91.67–100.00	75.00	20.00–80.00
	Non-responders	13	20.00 ^$^	−19.65–55.40	0.00 *	−25.00–45.00
Sex	Men	25	74.07	10–98.37	33.33	0.00–70.83
	Women	3	100.00	-	100.00	-
Treatment	Omeprazole	15	92.50 ^$^	65.71–100.00	60.00	0.00–80.00
	Esomeprazole	4	−57.14	−137.50–71.43	17.14	3.57–80.00
	Pantoprazole	5	20.00	−12.50–61.25	0.00	−50.00–36.67
	Lansoprazole	4	62.04	12.50–92.94	75.00	−39.58–95.83
Total		28	80.98	21.25–99.81	36.67	0.00–78.75

Data shown as median and interquartile range (IQR). PEC: peak eosinophils count; EREFS: endoscopic reference score. *: *p* < 0.05 compared to responders. ^$^: *p* < 0.004 compared to responders and to esomeprazole, pantoprazole, and lansoprazole treatment (threshold for significance adjusted after multiple comparisons).

**Table 5:** Percentage of PEC reduction and EREFS score reduction from baseline to after eight-week treatment according to genotypes and phenotypes.

Phenotype or Genotype		N	PEC Reduction %		EREFS Score Reduction %	
			Median	IQR	Median	IQR
*STAT6* g.18453G>C (rs12368672)	G/G	13	87.88	12.50–97.12	20.00	0.00–70.83
	G/C	13	74.07	35.00–100.00	66.67	7.14–91.67
	C/C	2	37.50	-	−75.00 *	-
*STAT6* g.27148G>A (rs167769)	G/G	11	91.67	56.25–99.23	33.33	0.00–100.00
	G/A	17	65.71	10.00–100.00	40.00	0.00–73.33
*STAT6* g.28741G>A (rs324011)	G/G	10	93.34	38.62–99.42	26.67	0.00–100.00
	G/A + A/A	18	69.89	10.00–100.00	45.00	0.00–70.00
*STAT6* g.37927C>T (rs841718)	C/C	3	56.25	-	0.00	-
	C/T	17	95.00	52.28–100.00	66.67	20.00–100.00
	T/T	8	56.44	5.00–91.89	7.14	0.00–57.50
*STAT6* g.38178C>T (rs3024974)	C/C	19	87.88	0.00–97.50	33.33	0.00–66.67
	C/T	9	74.07	55.40–100.00	66.67	−25.00–81.67
*STAT6* g.40823A>G (rs324015)	A/G	9	65.71	−19.65–98.75	66.67	10.00–100.00
	G/G	19	87.88	25.00–100.00	20.00	0.00–66.67
*STAT6* g.41214A>G (rs1059513)	A/A	21	90.04	52.28–100.00	50.00	0.00–77.50
	A/G	7	20.00	−100.00–99.23	14.29	0.00–100.00
CYP2C19	RM	9	57.89	0.00–93.94	40.00	0.00–73.33
	NM	13	91.67	5.36–99.62	50.00	0.00–87.50
	IM + PM	6	85.79	47.19–100.00	16.67	−12.50–87.50
CYP3A5	IM	4	95.45	63.83–99.81	83.33	16.67–100.00
	PM	24	69.89	5.00–99.38	26.67	0.00–72.92
*CYP3A4*	*1/*1	26	80.98	15.00–99.43	26.67	0.00–80.83
	*1/*22	2	75.00	-	70.83	-
*ABCB1* g.167964T>C (rs1128503)	T/T	6	95.02	47.19–100.00	0.00	−50.00–56.25
	T/C	14	61.80	−6.25–94.38	66.67	10.71–100.00
	C/C	7	74.07	0.00–99.23	33.33	0.00–83.33
*ABCB1* g.208920T>C (rs1045642)	T/T	5	56.25	−2.50–100.00	−15.28	−75.00–37.50
	C/T	15	87.88	54.55–100.00	66.67	20.00–100.00
	C/C	7	25.00	−14.29–99.23	14.29	0.00–40.00
*ABCB1* g.186947T>G/A (rs2032582)	T/T	4	77.87	59.85–97.51	75.00	12.50–100.00
	T/G	14	93.75	47.16–100.00	43.33	0.00–87.50
	G/G+G/A+A/A	10	35.00	−6.25–90.29	26.67 *	−56.25–61.67
TOTAL		28	80.98	78.56	36.67	78.75

Data shown as median and interquartile range (IQR). PEC: peak eosinophils count; EREFS: endoscopic reference score. RM: rapid metabolizer, NM: normal metabolizer, IM: intermediate metabolizer, PM: poor metabolizer. All patients showed the *STAT6* rs2598483 G/G genotype. * *p* < 0.05 compared to patients with *STAT6* rs12368672 G/C + G/G genotypes or with *ABCB1* rs2032582 T/T + T/G genotypes (nominally significant); no association reached the significance threshold after Bonferroni correction for multiple comparisons (*p* < 0.004).

**Table 6:** Genetic variants genotyped.

Gene	Genetic Variant	Allele/s Containing the Variant	TaqMan Assay ID(s)	RefSeq
*CYP2C19*	rs4244285	*2	C__25986767_70	NG_008384.3:g.24179G>A
	rs4986893	*3	C__27861809_10	NG_008384.3:g.22973G>A
	rs28399504	*4	C__30634136_10	NG_008384.3:g.5026A>G
	rs56337013	*5	C__27861810_10	NG_008384.3:g.95058C>T
	rs72552267	*6	C__27531918_10	NG_008384.3:g.17773G>A
	rs72558186	*7	C__30634127_10	NG_008384.3:g.24319T>A
	rs41291556	*8	C__30634130_30	NG_008384.3:g.17736T>C
	rs17884712	*9	C__25745302_30	NG_008384.3:g.17809G>A
	rs12248560	*17	C____469857_10	NG_008384.3:g.4220C>T
	rs12769205	*2,*35	AHWSL0R	NG_008384.3:g.17687A>G
*CYP3A4*	rs55785340	*2	C__30634204_10	NG_008421.1:g.20826T>C
	rs4986910	*3	C__27535825_20	NG_008421.1:g.28285T>C
	rs4646438	*6	C__32787140_40	NG_008421.1:g.22774dup
	rs28371759	*18	C__27859823_20	NG_008421.1:g.25183T>C
	rs35599367	*22	C__59013445_10	NG_008421.1:g.20493C>T
*CYP3A5*	rs776746	*3	C__26201809_30	NG_007938.2:g.12083A>G
	rs10264272	*6	C__30203950_10	NG_007938.2:g.19787G>A
	rs41303343	*7	C__32287188_10	NG_007938.2:g.32228dup
*ABCB1*	rs1045642	N/A	C___7586657_20	NG_011513.1:g.208920T>C
	rs2032582 ^$^	N/A	C_11711720D_40, C_11711720C_30	NG_011513.1:g.186947T>G/A
	rs1128503	N/A	C___7586662_10	NG_011513.1:g.167964T>C
*STAT6*	rs1059513	N/A	C___7480847_10	NG_021272.2:g.41214A>G
	rs324015	N/A	C____620398_10	NG_021272.2:g.40823A>G
	rs3024974	N/A	C__26439023_10	NG_021272.2:g.38178C>T
	rs841718	N/A	C___7480858_10	NG_021272.2:g.37927C>T
	rs324011	N/A	C____620399_10	NG_021272.2:g.28741G>A
	rs167769	N/A	C____620401_20	NG_021272.2:g.27148G>A
	rs2598483	N/A	C__15984966_10	NG_021272.2:g.24018G>A
	rs12368672	N/A	C__31186828_10	NG_021272.2:g.18453G>C

^$^ rs2032582 is a triallelic variant; therefore, two probes are necessary for its genotyping.

## Data Availability

The data that support the findings of this study are available from the corresponding author upon reasonable request.

## Supplementary Materials

The following supporting information can be downloaded at: https://www.mdpi.com/article/10.3390/ijms25073685/s1.
