# Supplementary material for: Impact of STAT6 Variants on the Response to Proton Pump Inhibitors and Comorbidities in Patients with Eosinophilic Esophagitis
Source: Int J Mol Sci. 2024 Mar 26;25(7):3685. doi: 10.3390/ijms25073685 (PMC11011338; doi:10.3390/ijms25073685)
Supplement: Supplementary file 1 [file ijms-25-03685-s001.zip › ijms-2901478-supplementary.pdf]

SUPPLEMENTARY MATERIAL

Supplementary Table S1. Genotype or phenotype and histological response cross-table.

Gene	Genotype/Phenotype	N	Responders	Non-responders
STAT6 g.41214A>G (rs1059513)	A/A	21	13 (61.91%)	8 (38.10%)
	A/G	7	2 (28.57%)	5 (71.43%)
STAT6 g.40823A>G (rs324015)	A/G	9	5 (55.56%)	4 (44.44%)
	G/G	19	10 (52.63%)	9 (47.37%)
STAT6 g.38178C>T (rs3024974)	C/C	19	10 (52.63%)	9 (47.37%)
	C/T	9	5 (55.56%)	4 (44.44%)
STAT6 g.37927C>T (rs841718)	C/C	3	0 (00.00%)	3 (100.00%)
	C/T	17	12 (70.59%)	5 (29.41%)
	T/T	8	3 (37.50%)	5 (62.5%)
STAT6 g.28741G>A (rs324011)	G/G	10	6 (60.00%)	4 (40.00%)
	G/A + A/A	18	9 (50.00%)	9 (50.00%)
STAT6 g.27148G>A (rs167769)	G/G	11	7 (63.64%)	4 (36.36%)
	G/A	17	8 (47.06%)	9 (52.94%)
STAT6 g.18453G>C (rs12368672)	G/G	13	6 (46.15%)	7 (53.85%)
	G/C	13	8 (61.54%)	5 (38.46%)
	C/C	2	1 (50.00%)	1 (50.00%)
CYP2C19	RM	9	4 (44.44%)	5 (55.56%)
	NM	13	7 (53.85%)	6 (46.15%)
	IM + PM	6	4 (66.67%)	2 (33.33%)
CYP3A5	IM	4	3 (75.00%)	1 (25.00%)
	PM	24	12 (50.00%)	12 (50.00%)
CYP3A4	*1/*1	26	14 (53.85%)	12 (46.15%)
	*1/*22	2	1 (50.00%)	1 (50.00%)
ABCB1 g.167964T>C (rs1128503)	T/T	6	3 (50.00%)	3 (50.00%)
	T/C	14	7 (50.00%)	7 (50.00%)
	C/C	7	4 (57.14%)	3 (42.86%)
ABCB1 g.208920T>C (rs1045642)	C/C	7	3 (42.86%)	4 (57.14%)
	C/T	15	9 (60.00%)	6 (40.00%)
	T/T	5	2 (40.00%)	3 (60.00%)
ABCB1 g.186947T>G/A (rs2032582)	T/T	4	2 (50.00%)	2 (50.00%)
	T/G	14	10 (71.43%)	4 (28.57%)
	G/G+G/A+A/A	10	3 (30.00%)	7 (70.00%)

Responders: <15 eosinophils/hpf. Non-responders: ≥15 eosinophils/hpf. RM: rapid metabolizer, NM: normal metabolizer, IM: intermediate metabolizer, PM: poor metabolizer.

Supplementary Table S2. Proton pump inhibitors and dose administered.

Active Principle	N	Dose
Omeprazole	14	40 mg/ 24h
	1	80 mg/ 24h
Esomeprazole	3	80 mg/ 24h
	1	40 mg/ 24h
Lansoprazole	5	60 mg/ 24h
Pantoprazole	5	80 mg/ 24h
